# Multidetector computed tomography in the evaluation of pediatric acute abdominal pain in the emergency department

**DOI:** 10.7603/s40681-016-0010-8

**Published:** 2016-05-06

**Authors:** Wei-Ching Lin, Chien-Heng Lin

**Affiliations:** 1 Department of Radiology, China Medical University Hospital, 404 Taichung, Taiwan; 2 Department of Biomedical Imaging and Radiological Science, College of Health Care, China Medical University, 404 Taichung, Taiwan; 3 Division of Pediatric Pulmonology, China Medical University Children’s Hospital, 404 Taichung, Taiwan

**Keywords:** Multidetector computed tomography, Abdominal pain, Children

## Abstract

The accurate diagnosis of pediatric acute abdominal pain is one of the most challenging tasks in the emergency department (ED) due to its unclear clinical presentation and non-specific findings in physical examinations, laboratory data, and plain radiographs. The objective of this study was to evaluate the impact of abdominal multidetector computed tomography (MDCT) performed in the ED on pediatric patients presenting with acute abdominal pain. A retrospective chart review of children aged <18 years with acute abdominal pain who visited the emergency department and underwent MDCT between September 2004 and June 2007 was conducted. Patients with a history of trauma were excluded. A total of 156 patients with acute abdominal pain (85 males and 71 females, age 1-17 years; mean age 10.9 ± 4.6 years) who underwent abdominal MDCT in the pediatric ED during this 3-year period were enrolled in the study. One hundred and eighteen patients with suspected appendicitis underwent abdominal MDCT. Sixty four (54.2%) of them had appendicitis, which was proven by histopathology. The sensitivity of abdominal MDCT for appendicitis was found to be 98.5% and the specificity was 84.9%. In this study, the other two common causes of nontraumatic abdominal emergencies were gastrointestinal tract (GI) infections and ovarian cysts. The most common etiology of abdominal pain in children that requires imaging with abdominal MDCT is appendicitis. MDCT has become a preferred and invaluable imaging modality in evaluating uncertain cases of pediatric acute abdominal pain in ED, in particular for suspected appendicitis, neoplasms, and gastrointestinal abnormalities.

## 1. Introduction

Acute abdominal pain is a common cause of pediatric visits to the emergency department (ED). Most cases are attributed to nonsurgical illnesses, and only a small fraction actually have an organic cause necessitating surgical intervention; the most common condition requiring surgery is acute appendicitis [1, 2]. Although medical history, a physical examination, and sonography can help physicians diagnose appendicitis out of various possible causes [3-5], physicians in the ED often order computed tomography (CT) when pediatric acute abdominal pain suggests appendicitis because CT has been proven to improve patient outcomes as reflected by lower negative laparotomy and perforation rates [6, 7]. In some studies, a dramatic increase in the use of CT in pediatric patients with abdominal pain has been observed in recent years without any change in the use of other imaging techniques, hospital admission rates, incidences of appendicitis, or severity of disease [8, 9].

The application of multidetector computed tomography (MDCT) has practical advantages for children in the ED because of its faster scanning time, better imaging quality (multiplanar reformation), lower sedation rate, and decreased radiation exposure.

The purpose of this study was to describe and evaluate the role of MDCT in pediatric acute abdominal pain in the ED.

## 2. Patients and methods

This was a retrospective registry-based cohort study of abdominal MDCT ordered in the ED by pediatric emergency physicians. From the ED CT log, consecutive patients aged <18 years old who had acute abdominal pain from September 2004 to June 2007 were identified. Trauma patients and patients who were discharged against medical advice were excluded.

Clinical information of age, sex, clinical presentation, sonography, CT results, and final diagnoses were obtained from charts. Sonographies were performed by a pediatric gastroenterologist. The decision to perform abdominal CTs was made by a pediatric emergency physician or a pediatric surgeon. The final diagnoses were recorded by reviewing the chart entries made by the attending pediatric emergency physicians after considering clinical history, laboratory data, and radiologic findings.

All CT scans were obtained with intravenous contrast enhanced using a 16- MDCT scanner (Lightspeed Ultra; GE Medical Systems, Milwaukee, Sis). All images were interpreted and recorded with a consensus between two radiologists. The CT criteria for acute appendicitis used by the reviewing radiologist included the following: a distended appendix greater than 7 mm in maximal diameter, appendiceal wall thickening and enhancement, an appendicolith, circumferential or focal apical cecal thickening, pericecal fat stranding, adjacent bowel wall thickening, focal or free peritoneal fluid, mesenteric lymphadenopathy, and intraperitoneal phlegmon, or an abscess [6].

All the excised appendices were sent for a pathology examination, and the final diagnosis of acute appendicitis was based on a histological examination. Negative appendectomy was defined as patients who underwent non-incidental appendectomy and the appendix was not found to be inflamed on pathologic examination.

The hospital’s institutional review board concurred that this retrospective study was a continuous quality improvement initiative for patient care and did not require informed consent.

## 3. Results

There were 156 ED computed tomography studies performed for acute abdominal pain in the pediatric emergency department during the 3-year period of this study. There were 85 males and 71 females, with an age range of 1-17 years (mean age, 10.9 ± 4.6 years).

### 3.1. Appendicitis

Among 118 children with suspected appendicitis, 72 were initially diagnosed as having appendicitis by CT, and 64 had the diagnosis of appendicitis confirmed later by pathologic results. Among the eight patients with false positive findings with MDCT, five of them did not have surgery because the family were reluctant, and their abdominal pain subsided after six to eight hours of observation. The remaining 3 patients had an operation: of these three patients, one’s pathology results revealed diverticulitis, and the other two had no inflammation in their excised appendices. All eight patients with false positive findings had an out-patient department follow-up and recovered completely. Forty-six patients who underwent MDCT revealed findings negative for appendicitis; however, one of them had persistent abdominal pain, and was later diagnosed with appendicitis that was proven by postoperative pathology. The rate of negative appendectomy was 9.7% (7/72). The sensitivity and specificity for diagnosing appendicitis by MDCT were found to be 98.5% and 84.9%, respectively. Moreover, ten patients with appendicitis had appendiceal perforation, and six of them had abscess formations. All abscess formations were detected *via* MDCT prior to surgery, but only one case of appendiceal perforation without abscess was detected *via* MDCT before the operation.

### 3.2. GI infection

The MDCT findings of 59 patients with acute abdominal pain showed thickening of the bowel walls, and five of them had ascites noted *via* MDCT. One of these patients underwent an appendectomy because of persistent right lower quadrant pain, and was diagnosed as having appendicitis from pathology after surgery. Another patient underwent exploratory laparotomy because of persistent rebounding pain, and was diagnosed as having gastric perforation. Among the 57 patients with GI infection, 12 of them were admitted to the ward for further treatment.

### 3.3. Gynecologic disease

Of the 12 children with gynecological diseases, eight had ovarian cysts, 3 had pelvic inflammatory disease, and 1 had ovarian torsion. Eight of these patients received sonographies initially, in which three were found to have fluid accumulation in the cul-de-sac and the other five had uncertain diagnoses. Subsequently, one ovarian torsion, one ruptured hemorrhagic ovarian, 4 ovarian cysts, and 2 pelvic inflammatory disease were detected *via* MDCT.

The patient with ovarian torsion and another with a ruptured hemorrhagic ovarian cyst both received surgeries. The remaining 10 patients were discharged after observation and medical treatment.

### 3.4. Bowel perforation

A total of 7 patients had bowel perforations, and 6 of them were diagnosed *via* MDCT. One patient who was diagnosed with inflammation of the bowel *via* MDCT pre-operatively actually had gastric perforation diagnosed after surgery. For this patient, two locations of extraluminal air bubbles were detected around the liver and stomach, and the perforations were associated with peptic ulcers diagnosed after a surgery. Another 4 locations of extraluminal air bubbles were found on the small bowel wall in 2 patients, the rectal wall in one patient, and the sigmoid wall in one patient. The perforation sites found *via* MDCT were compatible with the operative findings.

### 3.5. Neoplasms

There were five patients with intraabdominal masses detected initially via sonography, and MDCT was then performed for detailed evaluation. Their diagnoses were as follows: 2 teratomas, 1 lymphangioma, 1 hepatoblastoma, and 1 pheochromocytoma. All of them underwent operations, and their post-operative pathologies confirmed the pre-operative MDCT findings.

### 3.6. GI abnormalities

**Fig. 1:**
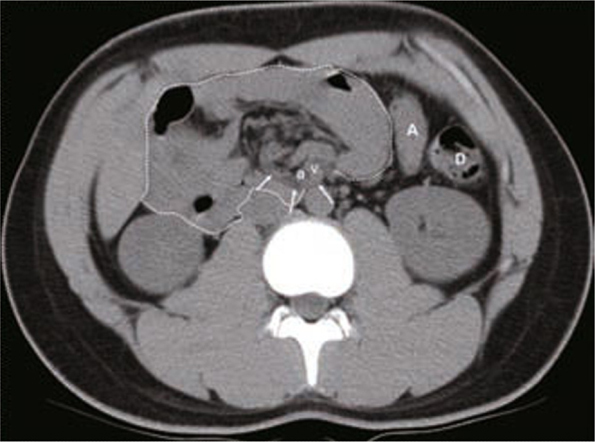
Axial MDCT reveals grouped small bowel loops (white dot line) in the right abdomen. The descending colon (D) is visible over the left abdomen but the ascending colon (A) is displaced to left side. Furthermore, the SMA (a) and SMV (v) are in the free edge of the sac; the looping of venous branches (white arrows) is also noted.

**Fig. 2:**
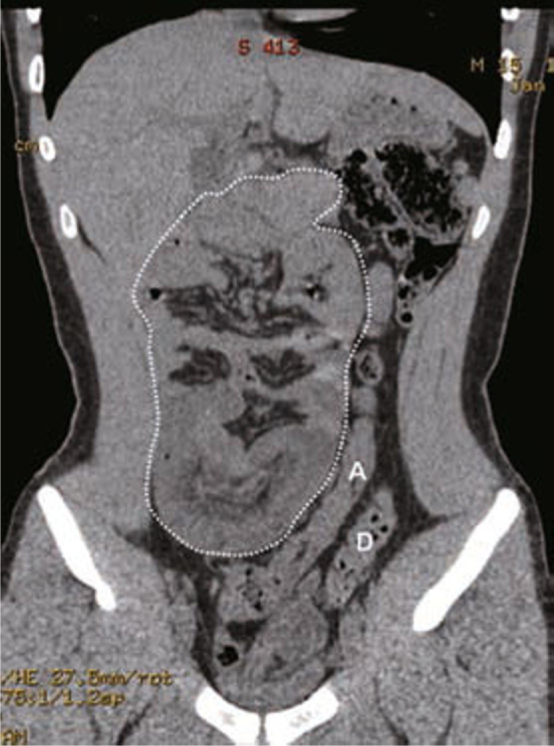
MDCT with coronal reformation demonstrates encapsulation of all small bowel loops in the right abdomen (white dot line) with displacement of the ascending colon (A) to the left abdomen parallel with the course of the descending colon (D).

A cystic mass was noted over the right lower quadrant of the abdomen in one patient via sonography, and a duplication cyst or an omental cyst was suspected after an abdominal MDCT. An exploratory laparotomy was performed and cystic duplication was proven by pathology. Abdominal MDCT of another patient revealed encapsulated small bowel loops on the right side of the abdomen and left-sided displacement of the ascending colon (Figures 1 & 2). A diagnosis of a right paraduodenal hernia was made preoperatively, which was subsequently proven correct on surgical exploration.

### 3.7. Others

The MDCT scan demonstrated colon wall thickening, a diverticulum and mild stranding of the pericolic fat in one patient, and a diagnosis of colonic diverticulitis was made. A huge renal cyst was found *via* MDCT in one patient, and hydronephrosis with urethral stones was detected *via* MDCT in another patient. Both of these patients did not receiv a sonography before MDCT.

**Table 1:** 

Final diagnoses	Case number
Appendicitis	64
GI infection	57
Ovarian cyst	8
Bowel perforations	7
Neoplasma	5
Pelvic inflammation diseases	3
Diverticulitis	2
Ovarian torsion	1
Hydronephrosis with urethral stone	1
Renal cyst	1
Duplication cyst	1
Paraduodenal hernia	1
Non specific abdominal pain (abdominal pain of unknown origin)	5

### 3.8. Abdominal pain of unknown origin

There were 5 patients with a final clinical diagnosis of abdominal pain of unknown origin. All of them had negative MDCT results and all were treated with intravenous hydration and several hours of observation in the ED. They were all later discharged after the symptoms of abdominal pain improved or abated.

The results of the final diagnoses are summarized in Table 1.

## 4. Discussion

The purpose of this study was to evaluate the role of MDCT in diagnosing acute abdominal pain in children visiting the ED. In 156 children with acute abdominal pain who underwent MDCT in the ED, the three most frequent diagnoses were appendicitis (41%), inflammatory bowel disease (36%), and ovarian cyst (5%). A previous study observed that the three most frequent diagnoses in 775 children with abdominal pain were gastrointestinal infection (65%), appendicitis (14%), and non-specific abdominal pain (13%) [5]. Another study by Reynolds and Jaffe demonstrated that the three most frequent diagnoses in 377 children with abdominal pain in an ED were nonspecific abdominal pain (36%), gastroenteritis (16%), and appendicitis (8%) [10]. Therefore, appendicitis is one of the most commonly observed, worrisome and serious conditions, and it necessitates surgery. Physicians in the ED should keep this in mind and rule out appendicitis for all pediatric patients with acute abdominal pain.

Imaging studies are not always needed in children with acute abdominal pain, and sonography should be used as the initial imaging modality for the evaluation of pediatric acute abdominal pain suspicious of appendicitis [11-13]. However, sonography is not always available to the pediatric gastroenterologist in our ED; moreover, the accuracy of a particular diagnosis is heavily dependent on the experience of the operator.

CT scans are always performed particularly when children have atypical presentations of appendicitis, abscess formations, or appendiceal perforation is suspected [14]. In a recent metaanalysis study, authors concluded that routine CT scans in all patients presenting with suspected appendicitis could reduce the rate of unnecessary surgery without increasing morbidity [15].

In our study, when children were suspected of having appendicitis by a physician in the ED, they were evaluated by the pediatric surgeon or, if a pediatric gastroenterologist was available, they underwent sonography first. In other words, a CT scan was performed when patients presented with atypical clinical findings, had equivocal sonographic results, or when the pediatric surgeon required a CT scan in order to make a decision. During our study period, appendicitis was diagnosed in an additional 243 patients; including 81 who underwent surgery without sonography and MDCT imaging studies, and another 162 patients who underwent sonography without MDCT scan. In our study, twelve patients with suspected appendicitis underwent sonography first and then MDCT. These patients underwent MDCT because of equivocal sonographic results. Subsequently, MDCT proved appendicitis in 8 patients, gastroenteritis in 3, and an ovarian cyst in 1.

Reviewing the detailed records of the false positive findings of MDCT, one of 5 patients had a distended appendix greater than 7 mm in diameter according to MDCT, and the other 4 had undirected signs such as cecal thickening, focal peritoneal fluid, mesenteric lymphadenopathy, or pericecal fat stranding. The sensitivity and specificity for diagnosing appendicitis by MDCT in the present study were 98.5% and 84.9%, respectively, whereas in the literature the average sensitivity and specificity of CT have been reported as 90.8% (range: 87%-100%) and 94.2% (89%-98%) respectively [11].

Other than appendicitis, GI infection is also a very common cause of acute abdominal pain of children who go to the ED, and it can easily be misdiagnosed as appendicitis. At the same time, GI infection is the most common diagnosis in cases of missed appendicitis [14-16]. In this situation, MDCT can be employed as a method to differentiate between these two etiologies of pediatric acute abdominal pain. Furthermore, MDCT can detect bowel perforations in patients with severe GI infection, when it will show intraperitoneal free air.

Sonography is the initial imaging study done to young females with acute abdominal pain. When sonography findings are too equivocal for a diagnosis, further evaluation with a CT scan is advocated [17]. Many gynecological disorders that cause acute abdominal pain (e.g., uterine disorders, ovarian disorders, pelvic inflammatory disease) demonstrate characteristic CT findings [17]. MDCT can provide useful information about abdominal fluid density differences in the pelvic cavity, and the presence of extravasated contrast material as an indicator of active bleeding [18, 19]. In one patient in the present study, sonography revealed a large amount of intrabdominal fluid and a hyperechogenic ovarian cyst, and a contrast-enhanced MDCT showed active extravasation from the ovarian cyst. Laparotomy was performed immediately in this patient and 1,300 ml of blood was aspirated from the peritoneal cavity. MDCT, thus, helped the emergency physician to render appropriate treatment for hemoperitoneum to this patient.

Bowel perforation is also a common cause of pediatric acute abdominal pain. There were 7 patients who had bowel perforations in this study, and six of them were diagnosed preoperatively through MDCT, which has already been established as the most valuable imaging technique for identifying the presence, site, and cause of a bowel perforation [20-22]. However, the amount of extraluminal air in appendiceal perforation is generally small or absent, usually no more than 1 or 2 ml, and a free pneumoperitoneum is very rare in patients with perforated appendicitis [22]. Therefore, in this study, only one case of appendiceal perforation without abscess formation was detected preoperatively by MDCT.

Abdominal neoplasms and gastrointestinal abnormalities are relatively uncommon causes of pediatric acute abdominal pain. In this study, there were five cases of neoplasms and two cases of GI anomalies. Most of the abdominal neoplasms or abnormalities had nonspecific findings on sonography, and they were identified more clearly by second-line examination of MDCT after contrast material injection, particularly in both the axial plane and the coronal plane images of the abdomen after reconstruction. In other words, it may be possible to make a precise pre-operative diagnosis of neoplasms and GI abnormalities through MDCT, and this is of great importance because it provides excellent anatomic details for correct diagnosis [23].

The main disadvantage of CT is the relatively high dose of radiation the patient receives. Furthermore, increased use of CT in children can raise their risk of cancer due to radiation exposure. The projected lifetime attributable risks of solid cancer are found to be higher for younger patients and females than for older patients and males; and they are also higher in patients who undergo CT scans of the abdomen/pelvis or spine than in patients who undergo other types of CT scans [24]. Besides, some patients have an allergic reaction after receiving contrast material injection, which was found to be the most common side effect of CT scans. In our study, no patients suffered from an allergic reaction to the contrast medium.

### 4.1. Limitations

This study has some limitations. The patients with abdominal pain without undergoing MDCT were not enrolled in our study because we focused on evaluating the impact of MDCT. The most important limitation, however, is that this is a retrospective review of medical records. The decision to use MDCT was not made by some pediatric emergency physicians, and their overuse of CT maybe criticized. The overuse of CT in ED is an important issue, and it may well be due to the clinician being afraid of malpractice suits or lacking experience, or else patient demand [25].

The small sample size of the study might have affected the sensitivity and specificity of MDCT to diagnose appendicitis. The follow-up information was insufficient except for those patients who were admitted to the wards or those who came back to the out-patient department. Lastly, this study only covered the experience of one hospital. A prospective study in multiple medical centers is required for a more thorough evaluation of using MDCT to diagnose abdominal pain in children.

## 5. Conclusion

Acute abdominal pain is a common complaint in children who are brought to the pediatric ED. Considering the risk of radiation from CT scans, MDCT is recommended in cases of pediatric abdominal pain with confusing presentation in the ED that cannot be diagnosed correctly after clinical data and sonography. The use of MDCT scans can offer greater accuracy as well as an ability to identify alternative diagnoses such as appendicitis, neoplasms and gastrointestinal abnormalities.
